# Lipase-mediated Baeyer–Villiger oxidation of benzylcyclopentanones in ester solvents and deep eutectic solvents

**DOI:** 10.1038/s41598-022-18913-2

**Published:** 2022-08-30

**Authors:** Marcelina Mazur, Tomasz Janeczko, Witold Gładkowski

**Affiliations:** Department of Food Chemistry and Biocatalysis, Wrocław University of Environmental and Life Sciences, Norwida 25, 50-375 Wrocław, Poland

**Keywords:** Biotechnology, Chemistry

## Abstract

This work presents the chemo-enzymatic Baeyer–Villiger oxidation of α-benzylcyclopentanones in ester solvents as well as deep eutectic solvents (DES). In the first part of the work the effect of selected reaction conditions on the reaction rate was determined. The oxidation process was most effective in ethyl acetate at 55 °C, with the use of lipase B from *Candida antarctica* immobilized on acrylic resin and UHP as oxidant. Ultimately, these preliminary studies prompted the development of an effective method for the implementation of lipase-mediated Baeyer–Villiger oxidation of benzylcyclopentanones in DES. The highest conversion was indicated when the oxidizing agent was a component of DESs (minimal DESs). The fastest conversion of ketones to lactones was observed in a mixture of choline chloride with urea hydrogen peroxide. In this case, after 3 days, the conversion of the ketones to lactones products exceeded 92% for all substrates. As a result, two new lactones were obtained and fully characterized by spectroscopic data.

## Introduction

Among others, lactones are an interesting group of compounds widely distributed in nature, as plant secondary metabolites. They exhibit many valuable biological properties, such as antimicrobial^1,2^, antifeedant^3,4^ or anti-inflammatory^5,6^. Among them, lactones containing an aromatic ring in the structure can also exhibit numerous biological properties, including anticancer activity^7–9^. One of the methods used to obtain compounds with a lactone moiety is the chemical Baeyer–Villiger (BV) oxidation. The substrates in this reaction are cyclic ketones, and the oxidation process can be carried out using organic peracids in traditional solvents such as toluene or dichloromethane. An alternative to the chemical synthesis is the use of enzymes, of which flavin-containing Baeyer–Villiger monooxygenases (BVMOs) are frequently mentioned^10,11^, but also occasionally lipases or acyltransferase^12–15^. The lack of coenzymes requirements, makes lipase application particularly interesting. Lipases, with the participation of hydrogen peroxide, catalyze the formation of the corresponding peroxyacids, which are then utilized in the oxidation of ketones to lactones. Typically, these reactions are carried out in conventional organic solvents such as ethyl acetate or toluene^13,14^. Alternatively, the use of deep eutectic solvents—DES as a medium, can be considered^16^. The benefits of using DESs come from the fact that they are generally attributed as "green" solvents, easily biodegradable, non-toxic, inexpensive, and easy to prepare^17–20^.

In recent years, there has been growing interest in the DESs as solvents with multiple applications for extraction^21,22^ chemical synthesis^23,24^ or biotransformation^25–27^. There are some examples of biotransformation reactions involving lipases as catalysts performed in DESs^28,29^. Nevertheless, there is not much data on the BV oxidation catalyzed in DESs. Wang et al. reported the successful BV oxidation of simple ketones such as cyclobutanone, cyclopentanone, or cyclohexanone to the corresponding lactones in several choline chloride-based DESs^30^. Compared with the traditional solvent, the authors noticed that the use of DESs limits the hydrolysis of the lactone to the corresponding hydroxy acid. Recently, Vagnoni also reported the BV oxidation in sugar-derived DES composed of glucose, fructose, sucrose and water (1:1:1:11) for model compounds like cyclopentanone and cyclohexanone^31^. The conversion for cyclohexanone after 64 h of reaction reached 75% and for cyclopentanone after 20 h only 15% of lactone was formed. Sieber and co-workers presented an interesting aspect of using so called “minimal DES” (mDES) in the epoxidation process^16^. The mDES consisted of choline chloride and urea hydrogen peroxide (UHP). Thus, the use of a suitable mDES provided both a solvent for the reaction and, at the same time, was a source of oxidizing agent. Therefore, it was no longer necessary to add an additional oxidant required for the C=C oxidation. For the epoxidation reaction studied, the mDES was considered the best solvent.

In this work, we were interested in obtaining lactones via a chemo-enzymatic process from α-benzylcyclopentanones. The reactions performed in DESs for this group of compounds are reported for the first time in the literature, and as a result of the experiments, the new lactone compounds were obtained. It is also important to note that we have achieved conversions comparable in both DES and ethyl acetate, which is traditionally used in the chemo-enzymatic BV reaction. The new approach to the synthesis was related to the use of mDES as an effective solvent for the process, as well as different ester solvents such as ethyl propionate, propyl acetate, ethyl benzoate, or methyl acetate. The effect of selected reaction conditions including temperature, type of lipase, or type of oxidant on the process efficiency was determined. The tested DESs were designed as conventional water-containing DESs as well as minimal DESs. The minimal DESs contained the oxidation agent, which was urea hydrogen peroxide (UHP) or hydrogen peroxide, as the DES component.

## Materials and methods

### Analysis

Thin Layer Chromatography (TLC, (silica gel on aluminum plates, DC-Alufolien Kieselgel 60 F254, Merck) and gas chromatography (GC, Agilent Technologies 6890 N instrument) were used to monitor the progress of the reactions. GC analysis was performed using an Agilent DB-5HT capillary column ((50%-phenyl)-methylpolysiloxane 30 m × 0.25 mm × 0.10 µm) and hydrogen as carrier gas. The following analysis conditions were applied: injector 250 °C, detector (FID) 250 °C, column temperature: 80–200 °C (25 °C × min^−1^), 200–300 °C (30 °C × min^−1^), 300 °C (3 min).

The purification of the products was carried out using the PuriFlash XS420Plus system and silica gel (column 30 µm Interchim, France).

The NMR spectra were performed on a JEOL 400 MHz Year Hold Magnet spectrometer and on a Brüker Avance II 600 MHz spectrometer (Bruker, Rheinstetten, Germany) in CDCl_3_ solution. The residual solvent signals (δ_H_ = 7.26, δ_C_ = 77.16) were used as references.

High-resolution mass spectra (HRMS) were registered on ESI-Q-TOF maXis impact spectrometer (Bruker Daltonics) using electron spray ionization (ESI) technique.

### Chemicals and enzymes

Octanoic acid (purity ≥ 99%), urea (98%), urea hydrogen peroxide (purity 97%), hydrogen peroxide 30 wt% solution in water were purchased from Sigma–Aldrich (USA). Choline chloride (purity 99%) was purchased from Acros organics (Belgium). Fructose (purity 99%) was purchased from Alfa Aesar (USA). Analytical grade chemicals: glucose, glycerol, sodium hydrogen carbonate, sodium thiosulphate, anhydrous magnesium sulphate, organic solvents were purchased from P.P.H. Stanlab (Poland), Chempur (Poland) and POCH (Poland) (Table S2). Lipase B acrylic resin from *Candida antarctica* (CAL-B AR, > 5000 U/g), Lipase B *Candida antarctica* immobilized on Immobead 150, recombinant from *Aspergillus oryzae* (CAL-B I150 ≥ 1800 U/g), lipase from *Rhizopus niveus* (RNL, ≥ 1.5 U/mg), lipase from *Rhizopus arrhizus* (RAL, ~10 U/mg) and Amano Lipase PS from *Burkholderia cepacia* (Amano PS, ≥ 30,000 U/ g) were purchased from Sigma-Aldrich (USA).

### Chemical synthesis

To a solution of ketone **1**, **3**, **5** (25 mmol) in anhydrous methylene chloride (30 mL) a solution of *meta*-chloroperoxybenzoic acid (*m*-CPBA) (0.5 g) in anhydrous methylene chloride (30 mL) was added. The mixture was stirred on a magnetic stirrer at room temperature for 2 days. After that, the reaction mixture was washed with Na_2_S_2_O_3_ and the layers were separated. The organic layer was washed with NaHCO_3_ followed by a saturated NaCl solution and dried with anhydrous MgSO_4_. The product was purified by flash chromatography and as a result three lactone compounds were obtained: 6-(4′-isopropylbenzyl)tetrahydropyran-2-one (**2**)—12 mg, isolated yields 21.4%; 6-(4′-methylbenzyl)tetrahydropyran-2-one (**4**)—25 mg, isolated yields 46.1%; 6-benzyltetrahydropyran-2-one (**6**)—15 mg, isolated yields 27.3%.

For comparison, the spectroscopic data of substrates and obtained products are given below.

#### 2-(4′-Isopropylbenzyl)cyclopentanone (**1**) (Fig. S1)

^1^H-NMR (400 MHz, CDCl_3_) δ: 1.24 (d, 6H, *J* = 6.9 Hz, (C*H*_3_)_2_ > CH), 1.56 (dtd, 1H, *J* = 12.4, 10.7, 6.6 Hz, one of CH_2_-3), 1.73 (m, 1H, one of CH_2_-4), 1.95 (m, 1H, one of CH_2_-4), 2.05–2.17 (m, 2H, one of CH_2_-3 and one of CH_2_-5), 2.29–2.39 (m, 2H, H-2 and one of CH_2_-5), 2.50 (dd, 1H, *J* = 13.9, 9.6 Hz, one of CH_2_-6), 2.88 (septet, 1H, *J* = 6.9 Hz, (CH_3_)_2_ > C*H*), 3.12 (dd, 1H, *J* = 13.9, 4.0 Hz, one of CH_2_-6), 7.08–7.16 (m, 4H, H-2′, H-3′, H-5′ and H-6′); ^13^C-NMR (100 MHz, CDCl_3_) δ: 20.7 (C-4), 24.2 and 24.2 (*C*H_3_)_2_ > CH), 29.4 (C-3), 33.8 ((CH_3_)_2_ > *C*H), 35.3 (C-6), 38.4 (C-5), 51.3 (C-2), 126.6 (C-3′ and C-5′), 128.9 (C-2′ and C-6′), 137.4 (C-1′), 146.8 (C-4′), 220.6 (C-1) HRMS: (ESI-TOF) m/z [M + Na]^+^ calcd for C_15_H_20_ONa 239.1406; found 239.1412.

#### 6-(4′-Isopropylbenzyl)tetrahydropyran-2-one (**2**) (Fig. S2)

Oily liquid, ^1^H-NMR (400 MHz, CDCl_3_) δ: 1.24 (d, 6H, *J* = 7.0 Hz, (C*H*_3_)_2_ > CH), 1.52 (m, 1H, one of CH_2_-5), 1.79 (m, 1H, one of CH_2_-4), 1.83–1.95 (m, 2H, one of CH_2_-4 and one of CH_2_-5), 2.43 (ddd, 1H, *J* = 17.7, 9.0, 7.0 Hz, one of CH_2_-3), 2.57 (m, 1H, one of CH_2_-3), 2.83 (dd, 1H, *J* = 13.9, 7.2 Hz, one of CH_2_-7), 2.88 (septet, 1H, *J* = 7.0 Hz, (CH_3_)_2_ > C*H*)), 3.06 (dd, 1H, *J* = 14.0, 5.9 Hz, one of CH_2_-7), 4.48 (dddd, 1H, *J* = 11.2, 7.3, 5.9, 3.0 Hz, H-6), 7.11–7.19 (m, 4H, Ar); ^13^C-NMR (100 MHz, CDCl_3_) δ: 18.5 (C-4), 24.1 ((*C*H_3_)_2_ > CH) 27.2 (C-5), 29.6 (C-3), 33.8 ((CH_3_)_2_ > *C*H) 41.8 (C-7), 81.3 (C-6), 126.7 (C-3′, C-5′), 129.6 (C-2′, C-6′), 133.8 (C-1′), 147.5 (C-4′), 171.9 (C-2). HRMS: (ESI-TOF) m/z [M + Na]^+^ calcd for C_15_H_20_O_2_Na 255.1356; found 255.1365.

#### 2-(4′-Methylbenzyl)cyclopentanone (**3**) (Fig. S3)

^1^H-NMR (400 MHz, CDCl_3_) δ: 1.55 (dtd, 1H, *J* = 12.5, 10.8, 6.7 Hz, one of CH_2_-3), 1.73 (dtdd, 1H, *J* = 12.8, 10.6, 8.4, 6.4 Hz, one of CH_2_-4), 1.95 (m, 1H, one of CH_2_-4), 2.05–2.14 (m, 2H, one of CH_2_-3 and one of CH_2_-5), 2.29–2.37 (m, 2H, H-2 and one of CH_2_-5), 2.32 (s, 3H, CH_3_-Ar), 2.51 (dd, 1H, *J* = 13.9, 9.5 Hz, one of CH_2_-6), 3.10 (dd, 1H, *J* = 13.9, 4.1 Hz, one of CH_2_-6), 7.04–7.11 (m, 4H, Ar); ^13^C-NMR (100 MHz, CDCl_3_) δ: 20.7 (C-4), 21.1 (CH_3_-Ar), 29.3 (C-3), 35.3 (C-6), 38.4 (C-5), 51.2 (C-2), 128.9 (C-2′ and C-6′), 129.2 (C-3′ and C-5′), 135.4 (C-4′), 137.0 (C-1′), 220.5 (C-1) HRMS: (ESI-TOF) m/z [M + Na]^+^ calcd for C_13_H_16_ONa 211.1093; found 211.1097.

#### 6-(4′-Methylbenzyl)tetrahydropyran-2-one (**4**) (Fig. S4)

Oily liquid, ^1^H-NMR (400 MHz, CDCl_3_) δ: 1.49 (m, 1H, one of CH_2_-5), 1.77 (m, 1H, one of CH_2_-4), 1.81–1.93 (m, 2H, one of CH_2_-5 and one of CH_2_-4), 2.32 (s, 3H, CH_3_-Ar), 2.42 (ddd, 1H, *J* = 15.6, 9.2, 6.8 Hz, one of CH_2_-3), 2.56 (m, 1H, one of CH_2_-3), 2.83 (dd, 1H, *J* = 14.0, 7.2 Hz, one of CH_2_-7), 3.05 (dd, 1H, *J* = 14.0, 6.0 Hz, one of CH_2_-7), 4.46 (dddd, 1H, *J* = 11.2, 7.2, 6.0, 2.8 Hz, H-6), 7.21–7.33 (m, 4H, Ar); ^13^C-NMR (100 MHz, CDCl_3_) δ: 18.5 (C-4), 21.2 (CH_3_-Ar), 27.1 (C-5), 29.6 (C-3), 41.8 (C-7), 81.3 (C-6), 129.4 and 129,6 (Ar), 133.4 (C-1′), 136.5 (C-4′), 171.8 (C-2). HRMS: (ESI-TOF) m/z [M + Na]^+^ calcd for C_13_H_16_O_2_Na 227.1043; found 227.1050.

#### 2-Benzylcyclopentanone (**5**) (Fig. S5)

^1^H-NMR (600 MHz, CDCl_3_) δ: 1.55 (m, 1H, one of CH_2_-3), 1.73 (m, 1H, one of CH_2_-4), 1.95 (m, 1H, one of CH_2_-4), 2.05–2.15 (m, 2H, one of CH_2_-3 and one of CH_2_-5), 2.31–2.39 (m, 2H, one of CH_2_-5 and H-2), 2.54 (dd, 1H, *J* = 13.9, 9.5 Hz, one of CH_2_-6), 3.15 (dd, 1H, *J* = 13.9, 4.1 Hz, one of CH_2_-6), 7.14–7.31 (m, 5H, Ar); ^13^C-NMR (151 MHz, CDCl_3_) δ: 20.7 (C-4), 29.3 (C-3), 35.8 (C-6), 38.4 (C-5), 51.2 (C-2), 126.3 (C-4′), 128.6 (C-5′, C-3′), 129.1 (C-2′, C-6′), 140.2 (C-1′), 220.4 (C-1); HRMS: (ESI-TOF) m/z [M + Na]^+^ calcd for C_12_H_14_ONa 197.0937; found 197.0941.

#### 6-Benzyltetrahydropyran-2-one (**6**) (Fig. S6)

Oily liquid, ^1^H-NMR (400 MHz, CDCl_3_) δ: 1.52 (m, 1H, one of CH_2_-5), 1.79 (m, 1H, one of CH_2_-4), 1.86–1.90 (m, 2H, one of CH_2_-5 and one of CH_2_-4), 2.43 (ddd, 1H, *J* = 17.7, 8.9, 6.9 Hz, one of CH_2_-3), 2.57 (m, 1H, one of CH_2_-3), 2.88 (dd, 1H, *J* = 13.9, 7.0 Hz, one of CH_2_-7), 3.09 (dd, 1H, *J* = 13.9, 5.8 Hz, one of CH_2_-7), 4.50 (dddd, 1H, *J* = 10.9, 7.0, 5.8, 2.9 Hz, H-6), 7.21–7.33 (m, 5H, Ar); ^13^C-NMR (100 MHz, CDCl_3_) δ: 18.5 (C-4), 27.2 (C-5), 29.6 (C-3), 42.2 (C-7), 81.2 (C-6), 127.0 (C-4′), 128.7 (C-3′, C-5′) 129.7 (C-2′, C-6′), 136.6 (C-1′), 171.8 (C-1). HRMS: (ESI-TOF) m/z [M + Na]^+^ calcd for C_12_H_14_O_2_Na 213.0886; found 213.0890.

### Preparation of DES

The components of DES mixtures were choline chloride (ChCl) as a hydrogen bond acceptor (HBA), urea (U), glycerol (Gly), glucose (Glu), fructose (Fru) and as a hydrogen bond donor (HBD). For DES preparation also water, 30% hydrogen peroxide or UHP was used (Table 1).

**Table 1:** List of the DES used in this study.

DES	1	2	3	4	5	6	7	8	9	10	11
HBD	Gly		Glu		U	Fru	UHP	U	Fru	Gly	Glu
Molar ratio HBA:HBD	1:2		2:1		1:2	1.9:1	1:2	1:2	1.9:1	1:2	2:1
% H_2_O^a^	10	50	30	50	10	10	0	0	0	0	0
% H_2_O_2_^a^	0	0	0	0	0	0	0	10	30	10	30

Hydrogen bond acceptor was choline chloride for all DES solutions.

^a^Water or hydrogen peroxide content (%, w/w).

The DES components (hydrogen bond donor and hydrogen bond acceptor and water or hydrogen peroxide) were placed in the 250 mL flask and stirred at 60 °C until a homogeneous and transparent liquid was formed.

### Lipase-mediated reaction condition

To a solution of ketone (25 µmol) in ester solvents (0.9 mL), lipase (5 mg) and UHP (5/10/20 mg) were added (Table 2). The tube was then sealed and placed on a ThermoMixer shaker at 800 rpm. After 1, 3, 6 and 10 days, the products were extracted three times with ethyl acetate. The combined extracts were washed with Na_2_S_2_O_3_ and dried over anhydrous magnesium sulphate. The reaction was monitored by gas chromatography. Control reactions were also carried out in optimized conditions. The reactions were prepared according to the procedure presented above, excluding lipase from the reaction mixture in the first case and UHP in the second.

**Table 2:** The conversion of ketone **1** to lactone **2** after 10 days of reaction performed in different ester solvents.

Temperature (°C)	Solvent	UHP (mg)	Lipase	Conversion after 10 days of incubation (%)
37	Ethyl acetate	10	CAL-B AR	19.5
45	Ethyl acetate	10	CAL-B AR	37.8
55	Ethyl acetate	10	CAL-B AR	47.2
55	Ethyl acetate	5	CAL-B AR	33.5
55	Ethyl acetate	20	CAL-B AR	63.6
55	Ethyl propionate	10	CAL-B AR	31.3
55	Propyl acetate	10	CAL-B AR	33.2
55	Ethyl benzoate	10	CAL-B AR	46.4
55	Methyl acetate	10	CAL-B AR	43
55	Ethyl acetate	10	RNL	0
55	Ethyl acetate	10	RAL	8.7
55	Ethyl acetate	10	CAL-B I150	31.1
55	Ethyl acetate	10	Amano PS	6.8
55	Ethyl acetate	20	CAL-B AR ^a^	99 (3 days)
55	Ethyl acetate	20	–	0 (3 days)^b^
55	Ethyl acetate	0	CAL-B AR ^a^	0 (3 days)^c^

^a^Doubling of the amount of lipase.

^b^Control without lipase.

^c^Control without UHP.

For water-containing DESs, ketone (25 µmol), lipase CAL-B AR (5 mg), octanoic acid (50 µL) and UHP (10 mg) were added. Subsequent steps were carried out as for experiments with organic solvents.

Minimal DESs experiments. To a solution of ketone (25 µmol) in DES (0.9 mL), lipase CAL-B AR (5 mg) and octanoic acid (50 µL) were added. Depending on the DES tested, different oxidants were used (Table 1). The next steps were carried out as described for the organic solvent. Control reactions were also carried out. The reactions were prepared according to the procedure presented above, excluding lipase from the reaction mixture in the first control, octanoic acid in the second, and the oxidizing agent in the third. Macroscopic changes in the appearance of individual DESs were also monitored during the process. No separation or precipitation of the mixture components was observed. No color change was observed (except for fructose-containing DES) and all DESs remained clear (Figs. S7, S8).

### Ethical approval

This article does not contain any studies with human participants or animals performed by any of the authors.

## Results and discussion

The benzyl derivatives of cyclopentanone (**1**, **3**, **5**) (Figs. S1, S3, S5) were subjected to chemical Baeyer–Villiger oxidation. Products containing lactone ring (**2**, **4**, **6**) (Figs. S2, S4, S6) served as standard used to monitor chemo-enzymatic reactions. The spectral data of cyclic ketones (**1**, **3**, **5**) and 6-benzyltetrahydropyran-2-one (**6**) are in accordance with the literature^32–34^. The two other lactones, with *p*-methyl (**4**) and *p*-isopropyl (**2**) substituents, were not previously described. The NMR spectra of these compounds are very similar to those of lactone **6**. In comparison to the spectrum of ketones, one can see a clear difference in the chemical shifts of the signal from methine protons. Those signals in ketones spectra are located at 2.29–2.39 ppm, whereas in lactones spectra they are shifted to 4.46 ppm (**4**) or 4.48 ppm (**6**). This is the result of the deshielding effect of the oxygen atom in the lactone ring. The same deshielding effect can be observed in the ^13^C NMR spectrum. Signals from C-2 carbon atoms are present at 51 ppm in ketones spectra, while signals from those carbons in the lactone ring (C-6) are shifted to 81 ppm. The signals of carbonyl C atoms in the ketones spectra are nearly identical and are shifted to 220 ppm, which is characteristic for five-membered cyclic ketones. In the spectra of lactones they absorb at higher field and their chemical shift value (171 ppm) is characteristic for σ-lactone ring. The spectroscopic data indicated the regioselectivity of the incorporation of an oxygen atom into the cyclopentanone ring. In accordance with the reaction mechanism, the oxygen atom is inserted between the higher substituted α-carbon atom and carbonyl group.

Cyclic ketones were the substrates for chemo-enzymatic oxidation using lipases as catalysts. Lipases, with the participation of hydrogen peroxide present in the reaction, catalyzed the oxidation of organic acids or esters to the corresponding peroxyacids. The peroxyacid formed "in situ" is capable of oxidizing ketones to lactones.

The influence of several factors on the oxidation process was determined (Table 2). First, the effects of temperature on oxidation yield were investigated. In the first experiments, reactions were carried out with CAL-B lipase immobilized on acrylic resin (CAL-B AR) in ethyl acetate using a urea-hydrogen peroxide complex at 37, 45 and 55 °C. As the temperature increased, the reaction proceeded faster and the best oxidation results were obtained at 55 °C. To check the effect of the solvent, the reactions were performed in different ester solvents. The highest conversion (46–47%) was obtained in ethyl acetate and ethyl benzoate. However, because of the lower volatility and higher price of ethyl benzoate, ethyl acetate was chosen as the optimal solvent. The possibility of using different lipases was also investigated. Apart from CAL-B AR, the enzymes used as catalysts were commercially available: lipase from *Rhizopus niveus* (RNL), lipase from *Rhizopus arrhizus* (RAL), lipase B from *Candida antarctica* immobilized on Immobead (CAL-B I150), Amano PS lipase from *Burkholderia cepacia*. The best catalyst for the oxidation process was CAL-B AR lipase. The second best biocatalyst appeared to be lipase CAL-B I150, but for this enzyme the conversion did not exceed 32% after 10 days of the process. In the case of lipase from *Rhizopus niveus,* no formation of reaction product was observed. On this basis, CAL-B AR lipase was selected as an effective biocatalyst in further stages of the study. Although examples of the use of different lipases in the BV oxidation reaction can be found in the literature, studies on the use of different types of esters as peracid precursors are not widely reported. In recent years, the literature has described the use of an increasing number of biocatalysts, including those genetically modified, but CAL-B is still one of the most effective for this process^14,30,35^. The results presented in this paper also show that among the commercially available lipases tested, the CAL-B is the most effective one. The effect of the oxidant on the reaction rate was also taken into account. Therefore, in the next step, the effect of the amount of UHP and lipase added was checked. Doubling the amount of lipase combined with an increase in the amount of added UHP allowed to achieve a conversion rate of 99% after only 3 days of the process.

**Figure 1:**
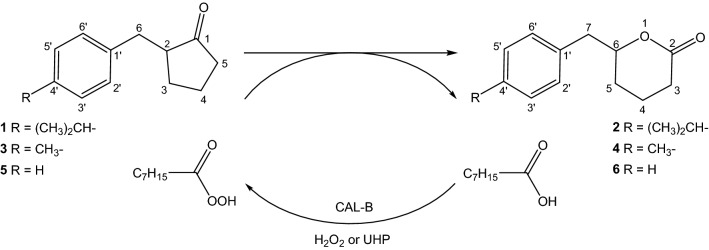
Chemo-enzymatic Baeyer–Villiger oxidation of α-benzylcyclopentanones in different mDESs.

An important aspect was to evaluate the possibility of using deep eutectic solvents as alternative solvents in the oxidation process. Reactions were carried out in DESs 1–6 based on choline chloride and urea, glycerol, glucose and fructose with the addition of water (Table 1). Initially, in the reactions of chemo-enzymatic oxidation, UHP was added and octanoic acid was used as a peroxyacid precursor. Unfortunately, no conversion was observed, even after 10 days of running the process. Therefore, the possibility of using minimal DES, in which the oxidizing agent is also a component of the eutectic mixture, was tested. Two oxidizing agents UHP and a 30% hydrogen peroxide solution were tested (Fig. 1).

mDESs consisted of choline chloride and UHP (mDES 7) or choline chloride and urea (mDES 8), glycerol (mDES 10), fructose (mDES 9) or glucose (mDES 11), with the appropriate addition of the hydrogen peroxide solution (Table 1). The conversion of substrates strongly depended on the type of HBD (Table 3). The most efficient was the solvent composed of choline chloride and UHP (mDES 7). After only 3 days of the process, the conversion of ketone with *p*-isopropylbenzyl (**1**) and *p*-methylbenzyl (**3**) substituent was 99%, while for the oxidation of benzylcyclopentanone (**5**) a conversion of 92% was observed. When hydrogen peroxide containing DESs were used, the reaction occurred most effectively in a mixture of choline chloride and urea (mDES 8). In this case, the conversion after 3 days of the process was 98% for ketone **1**, 95% for ketone **3** and 91% for ketone **5**. Good yields, exceeding 70% after 3 days, were also obtained by performing the reaction in DESs containing sugars: glucose (mDES 11) and fructose (mDES 9). The lowest conversion, which did not exceed 10% after 6 days, was observed in the case of application of the mixture of choline chloride and glycerol (mDES 10).

**Table 3:** The conversion (according to GC) of ketones **1**, **3** and** 5** to corresponding lactones in different DES medium.

mDES from Table1	Time days	Conversion (%)						Control reactions^a^	
		Ketone **1**	Lactone **2**	Ketone **3**	Lactone **4**	Ketone **5**	Lactone **6**	Ketone **1,3,5**	Lactone **2,4,6**
7	1	59.8	40.1	73.2	26.8	88.6	11.4	100	0
	2	11.1	88.9	9.9	90.1	47.6	52.4	100	0
	3	1.0	99.0	0.9	99.1	7.9	92.1	100	0
	6	0	100	0	100	0	100	100	0
8	1	26.0	74.0	41.7	58.3	68.1	31.9	100	0
	2	2.0	98.0	8.7	91.3	15.8	84.2	100	0
	3	1.9	98.1	5.0	95.0	9.3	90.7	100	0
	6	1.3	98.7	4.7	95.3	7.0	93.0	100	0
9	1	25.7	74.3	65.1	34.9	60.2	39.8	100	0
	2	18.3	81.7	36.0	64.0	31.4	68.6	100	0
	3	16.5	83.5	22.8	77.2	29.1	70.9	100	0
	6	12.1	87.9	19.1	80.9	28.0	72.0	100	0
10	1	97.4	2.6	95.4	4.5	95.0	5.0	100	0
	2	94.0	6.0	94.9	5.1	95.8	4.2	100	0
	3	94.0	6.0	92.5	7.5	95.5	4.5	100	0
	6	76.4	23.6	91.9	8.1	94.4	5.6	100	0
11	1	10.7	89.3	35.2	64.8	49.1	50.9	100	0
	2	9.4	90.6	16.2	83.8	33.8	66.2	100	0
	3	6.8	93.2	14.6	85.4	29.4	70.6	100	0
	6	3.6	96.4	7.4	92.6	21.3	78.7	100	0

^a^Control reactions were carried out by excluding respectively lipase, octanoic acid and oxidant from the reaction mixture.

With the growing public's environmental awareness, ecological techniques are also increasingly being introduced as part of chemical processes. Therefore, green techniques have been explored with the development of green chemistry. Consequently, the use of more environmentally friendly and economic solvents instead of hazardous solvents is one of the most relevant aspects in the pursuit of greener technologies^17^. DESs proposed as solvents for chemo-enzymatic BV oxidation processes are composed of biological components, such as choline chloride, sugars, glycerol, or urea, and possess many advantages, such as low cost, easy preparation, and biodegradability. The chemo-enzymatic BV reaction is impossible to carry out without the addition of the oxidant, and hydrogen peroxide is decomposed to yield only oxygen and water, which means it is one of the cleanest, most versatile oxidative agents. Therefore, the addition of hydrogen peroxide in its pure form or adduct of hydrogen peroxide with urea (UHP) seems to be sustainable solution for this type of process.

Although chemo-enzymatic BV oxidation has been known since the 1990s^36^, new methods are still being developed to increase the efficiency of the process or its environmental friendliness. Recently, Szelwicka et al. proposed a process in which multi-walled carbon nanotubes were applied as a support for ionic liquids which were anchored to nanotubes covalently by amide or imine bonds^35^. Although the process efficiency tested on 2-adamantanone is very promising, it is not a very accessible method at that moment, and requires an advanced immobilization process. In contrast, the use of minimal DESs composed of readily available and inexpensive ingredients is much easier to apply in standard laboratory practice.

## Conclusions

In this study, we evaluated the possibility of the application of various esters and deep eutectic solvents as a media for the Baeyer–Villiger type chemo-enzymatic oxidation of cyclic ketones with benzyl substituents. Among the tested variants of reactions carried out in ester solvents, the most advantageous was the use of ethyl acetate with lipase CAL-B AR at 55 °C. Other effective solvents were minimal DESs, which contained the oxidation agent (UHP or hydrogen peroxide) as the DES component. The conversion was directly related to the type of HBD used in the solvent. It was advantageous to use urea or UHP in the mDES mixture. Sufficient conversion was also achieved when sugars were used as HBD components. What is also worth pointing out that, generally compared to unsubstituted ketones, the large substituent in the α position lowered the reaction rate^13^. Therefore, it is worth highlighting that for the tested α-benzylcyclpoentanones, the conversion obtained in DESs was comparable to that observed in ethyl acetate as well as the conversion obtained by Wang and al. for unsubstituted cyclopentanone^30^. Additionally, as a result of experiments in this paper, two new compounds with *p*-methylbenzyl and *p*-isopropylbenzyl substituents were obtained and characterized by spectroscopic data. In summary, our work presents the first successful attempt on the implementation of lipase-mediated Baeyer–Villiger oxidation of α-benzylcyclopentanones in deep eutectic solvents.

## Supplementary Information

Supplementary Information.

## Data Availability

All major data generated and analyzed in this study are included in this manuscript and its supplementary information files.

## References

[1] Wińska K (2018). Antimicrobial activity of new bicyclic lactones with three or four methyl groups obtained both synthetically and biosynthetically. J. Saudi Chem. Soc..

[2] Pomini AM, Marsaioli AJ (2008). Absolute configuration and antimicrobial activity of acylhomoserine lactones. J. Nat. Prod..

[3] Mazur M (2016). Lactones 46: Synthesis, antifeedant and antibacterial activity of γ-lactones with a p-methoxyphenyl substituent. Pest Manag. Sci..

[4] Mazur M (2014). Lactones 43: New biologically active lactones: β-cyclocitral derivatives. Pest Manag. Sci..

[5] Wu ZN (2017). Sesquiterpene lactones from *Elephantopus mollis* and their anti-inflammatory activities. Phytochemistry.

[6] Abe AE (2015). Anti-inflammatory sesquiterpene lactones from tithonia diversifolia trigger different effects on human neutrophils. Rev. Bras. Farmacogn..

[7] Kamizela A (2018). Synthesis, characterization, cytotoxicity, and antibacterial properties of trans-γ-halo-δ-lactones. Chem. Open.

[8] Pawlak A (2017). A novel canine B-cell leukaemia cell line: Establishment, characterisation and sensitivity to chemotherapeutics. Vet. Comp. Oncol..

[9] Pawlak A (2018). Enantiomeric trans β-aryl-δ-iodo-γ-lactones derived from 2,5-dimethylbenzaldehyde induce apoptosis in canine lymphoma cell lines by downregulation of anti-apoptotic Bcl-2 family members Bcl-xL and Bcl-2. Bioorg. Med. Chem. Lett..

[10] Bučko M (2016). Baeyer-Villiger oxidations: Biotechnological approach. Appl. Microbiol. Biotechnol..

[11] Gniłka R, Szumny A, Białońska A, Wawrzeńczyk C (2012). Lactones 39: 1 chemical and microbial synthesis of lactones from (-)-α- and (+)-β-thujone. Phytochem. Lett..

[12] Drozdz A, Hanefeld U, Szymańska K, Jarzȩbski A, Chrobok A (2016). A robust chemo-enzymatic lactone synthesis using acyltransferase from Mycobacterium smegmatis. Catal. Commun..

[13] Ríos MY, Salazar E, Olivo HF (2007). Baeyer–Villiger oxidation of substituted cyclohexanones via lipase-mediated perhydrolysis utilizing urea–hydrogen peroxide in ethyl acetate. Green Chem..

[14] Drozdz A, Chrobok A (2016). Chemo-enzymatic Baeyer–Villiger oxidation of 4-methylcyclohexanone via kinetic resolution of racemic carboxylic acids: Direct access to enantioenriched lactone. Chem. Commun..

[15] Drozdz A (2013). The chemo-enzymatic Baeyer–Villiger oxidation of cyclic ketones with an efficient silica-supported lipase as a biocatalyst. Appl. Catal. A Gen..

[16] Ranganathan S, Zeitlhofer S, Sieber V (2017). Development of a lipase-mediated epoxidation process for monoterpenes in choline chloride-based deep eutectic solvents. Green Chem..

[17] Pätzold M (2019). Deep eutectic solvents as efficient solvents in biocatalysis. Trends Biotechnol..

[18] Perna FM, Vitale P, Capriati V (2020). Deep eutectic solvents and their applications as green solvents. Curr. Opin. Green Sustain. Chem..

[19] Paiva A (2014). Natural deep eutectic solvents: Solvents for the 21st century. ACS Sustain. Chem. Eng..

[20] Yu D, Xue Z, Mu T (2021). Eutectics: Formation, properties, and applications. Chem. Soc. Rev..

[21] Grudniewska A, Popłoński J (2020). Simple and green method for the extraction of xanthohumol from spent hops using deep eutectic solvents. Sep. Purif. Technol..

[22] Grudniewska A (2018). Enhanced protein extraction from oilseed cakes using glycerol-choline chloride deep eutectic solvents: A biorefinery approach. ACS Sustain. Chem. Eng..

[23] Mota-Morales JD (2018). Free-radical polymerizations of and in deep eutectic solvents: Green synthesis of functional materials. Prog. Polym. Sci..

[24] Di Carmine G, Abbott AP, D’Agostino C (2021). Deep eutectic solvents: Alternative reaction media for organic oxidation reactions. React. Chem. Eng..

[25] CvjetkoBubalo M, Mazur M, Radošević K, RadojčićRedovniković I (2015). Baker’s yeast-mediated asymmetric reduction of ethyl 3-oxobutanoate in deep eutectic solvents. Process Biochem..

[26] Pavoković D, Košpić K, Panić M, RadojčićRedovniković I, CvjetkoBubalo M (2020). Natural deep eutectic solvents are viable solvents for plant cell culture-assisted stereoselective biocatalysis. Process Biochem..

[27] Panić M, Delač D, Roje M, RadojčićRedovniković I, CvjetkoBubalo M (2019). Green asymmetric reduction of acetophenone derivatives: *Saccharomyces cerevisiae* and aqueous natural deep eutectic solvent. Biotechnol. Lett..

[28] Panić M (2021). Development of environmentally friendly lipase-catalysed kinetic resolution of (R, S)-1-phenylethyl acetate using aqueous natural deep eutectic solvents. Process Biochem..

[29] Juneidi I, Hayyan M, Hashim MA, Hayyan A (2017). Pure and aqueous deep eutectic solvents for a lipase-catalysed hydrolysis reaction. Biochem. Eng. J..

[30] Wang XP (2017). Engineering a lipase B from *Candida **antactica* with efficient perhydrolysis performance by eliminating its hydrolase activity. Sci. Rep..

[31] Vagnoni M (2021). Lipase catalysed oxidations in a sugar-derived natural deep eutectic solvent. Biocatal. Biotrans..

[32] Li Q (2014). Enantioselective hydrogenation of the double bond of exocyclic α, β-unsaturated carbonyl compounds catalyzed by iridium/H8-BINOL-derived phosphine-oxazoline complexes. Asian J. Org. Chem..

[33] Kaku H, Ito M, Horikawa M, Tsunoda T (2018). Deracemization of α-monosubstituted cyclopentanones in the presence of a TADDOL-type host molecule. Tetrahedron.

[34] Rios MY, Salazar E, Olivo HF (2008). Chemo-enzymatic Baeyer–Villiger oxidation of cyclopentanone and substituted cyclopentanones. J. Mol. Catal. B Enzym..

[35] Szelwicka A (2021). Chemo-enzymatic Baeyer–Villiger oxidation facilitated with lipases immobilized in the supported ionic liquid phase. Materials.

[36] Lemoult SC, Richardson PF, Roberts SM (1995). Lipase-catalysed Baeyer–Villiger reactions. J. Chem. Soc. Perkin Trans..

