# Supplementary material for: Lipase-mediated Baeyer–Villiger oxidation of benzylcyclopentanones in ester solvents and deep eutectic solvents
Source: Sci Rep. 2022 Aug 30;12:14795. doi: 10.1038/s41598-022-18913-2 (PMC9427991; doi:10.1038/s41598-022-18913-2)
Supplement: Supplementary file 1 — Supplementary Information. [file 41598_2022_18913_MOESM1_ESM.pdf]

Scientific Reports

Supplementary Materials

Lipase-mediated Baeyer-Villiger oxidation of benzylcyclopentanones in esters solvents and deep eutectic solvents

Marcelina Mazur[1,*], Tomasz Janeczko[1], Witold Gładkowski[1]

[1]Department of Food Chemistry and Biocatalysis, Wrocław University of Environmental and Life Sciences, Norwida 25, 50-375 Wrocław, Poland

*Correspondence: Dr. Marcelina Mazur marcelina.mazur@upwr.edu.pl; Tel/Fax.: +48-713205197

Number of pages: 11

8 Figures, 2 Table

List of Supplementary Materials.

Figure S1. ^{1}H-NMR and ^{13}C-NMR spectrum of ketone 1.

Figure S2. ^{1}H-NMR and ^{13}C-NMR spectrum of lactone 2.

Figure S3. ^{1}H-NMR and ^{13}C-NMR spectrum of ketone 3.

Figure S4. ^{1}H-NMR and ^{13}C-NMR spectrum of lactone 4.

Figure S5. ^{1}H-NMR and ^{13}C-NMR spectrum of ketone 5.

Figure S6. ^{1}H-NMR and ^{13}C-NMR spectrum of lactone 6.

Figure S7. The DESs after 3 days at 25 and 55°C.

Figure S8. The DESs after 6 days at 25 and 55°C.

Table S1. Comparison of literature data showing BV-type oxidation of cyclic ketones.

Table S2. The list of chemicals used in the experiments.

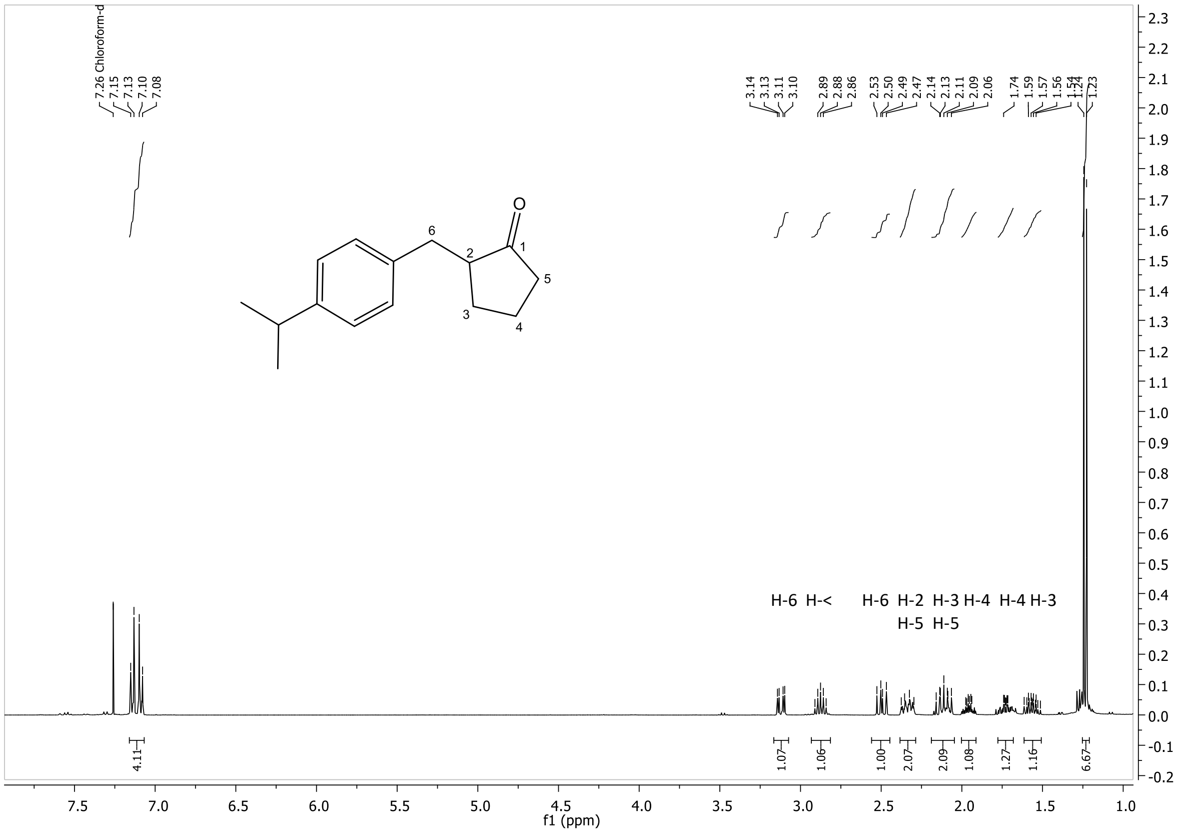

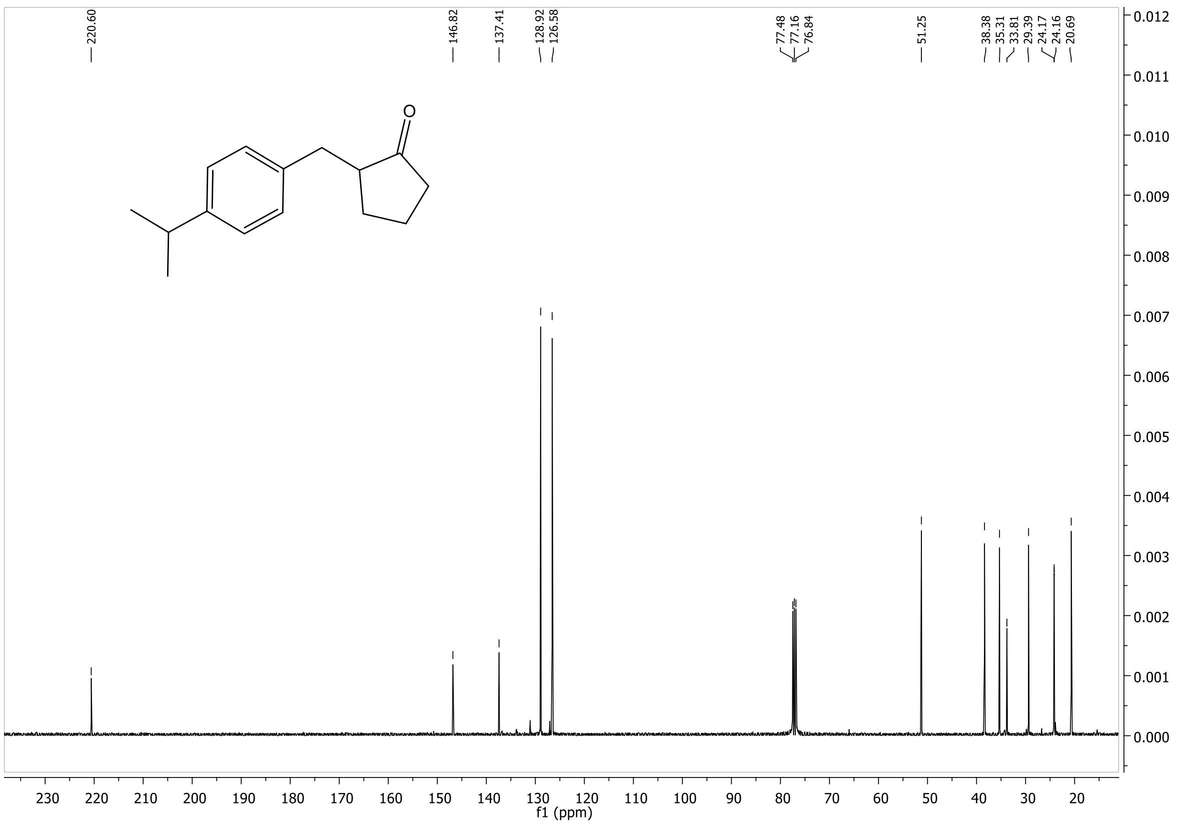

Figure S1: ^{1}H-NMR and ^{13}C-NMR spectrum of ketone 1.

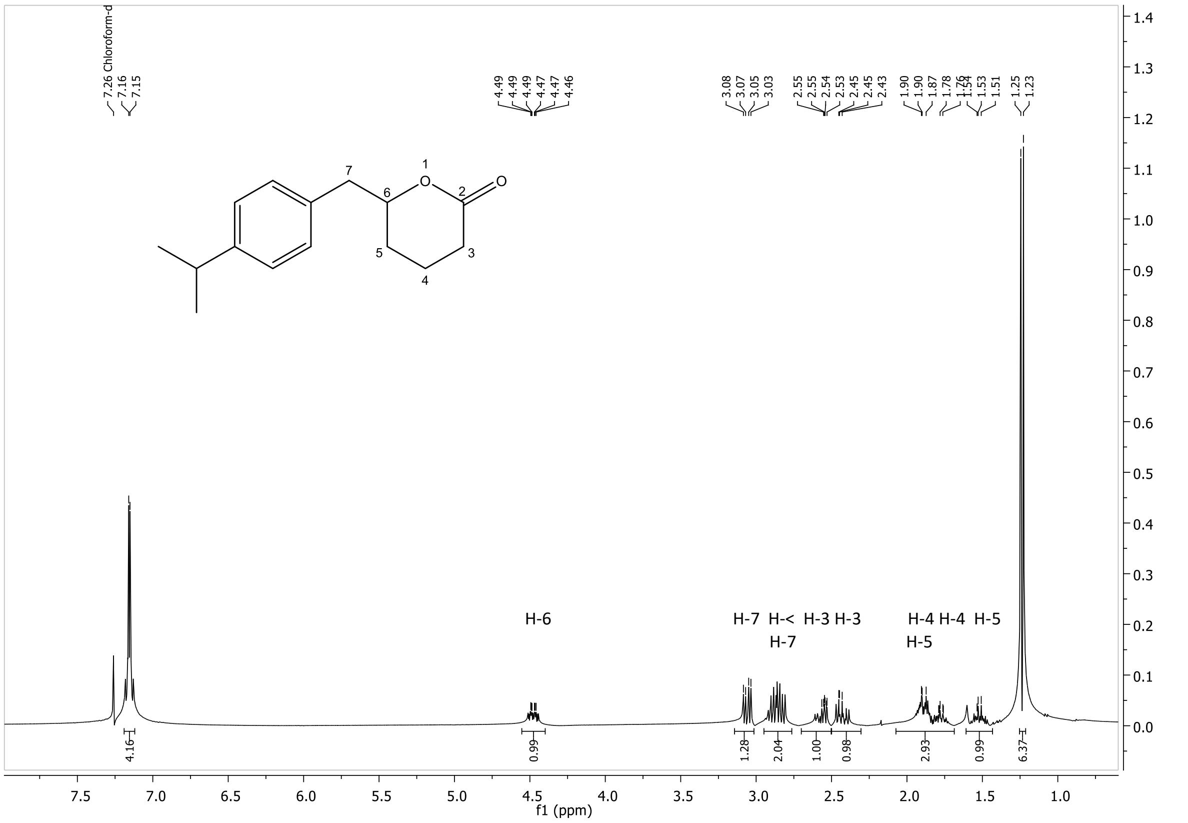

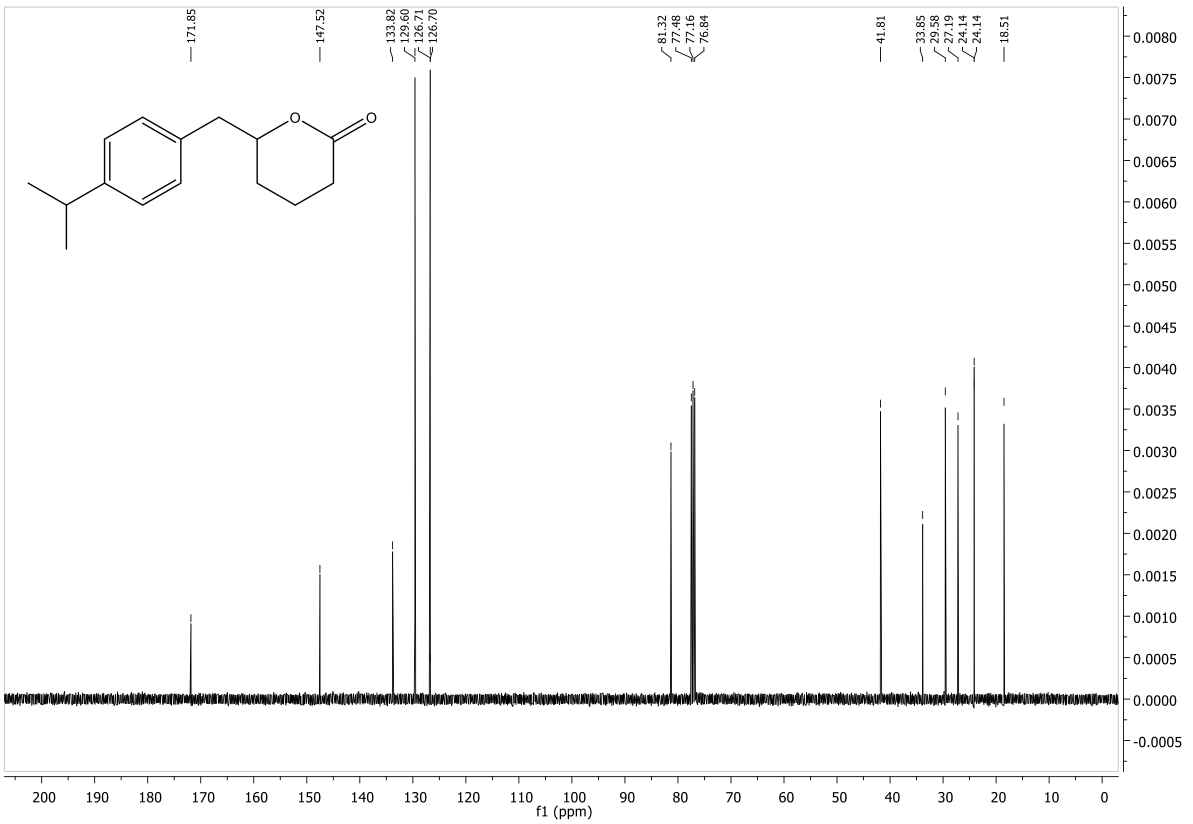

Figure S2: ^{1}H-NMR and ^{13}C-NMR spectrum of lactone 2.

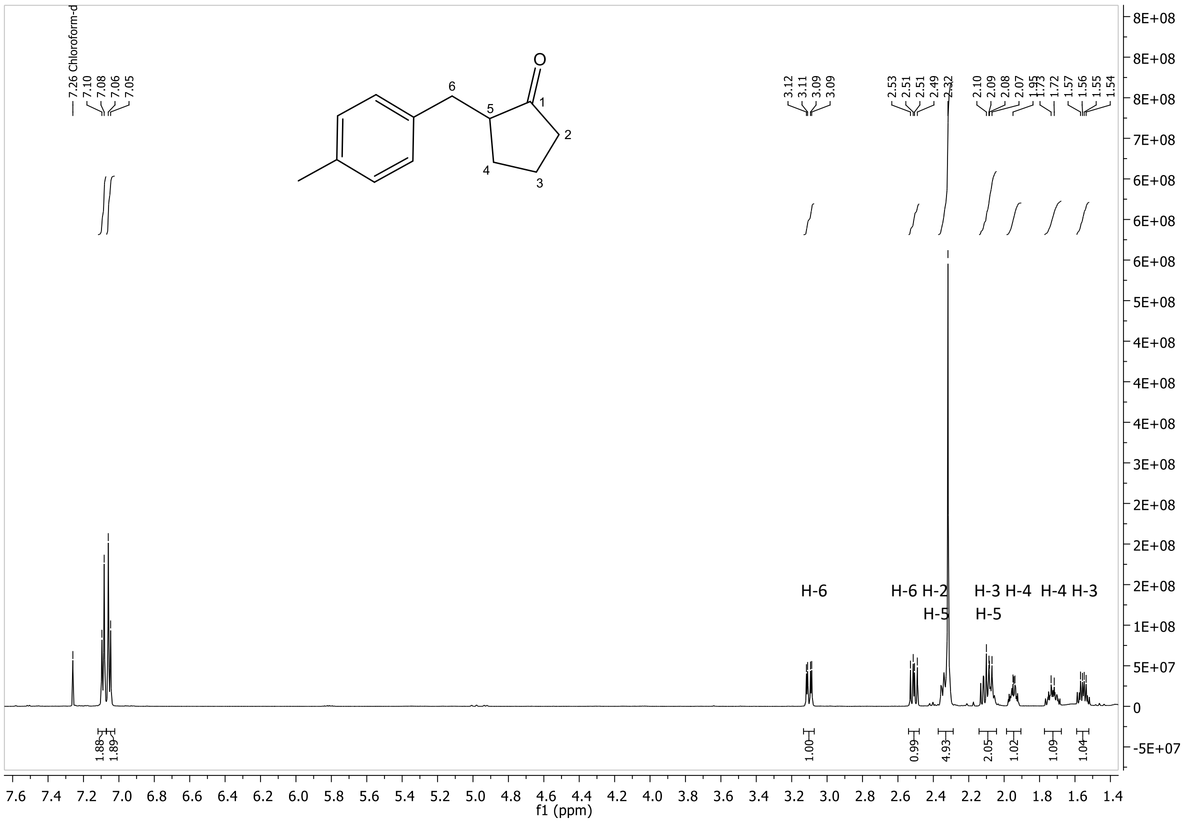

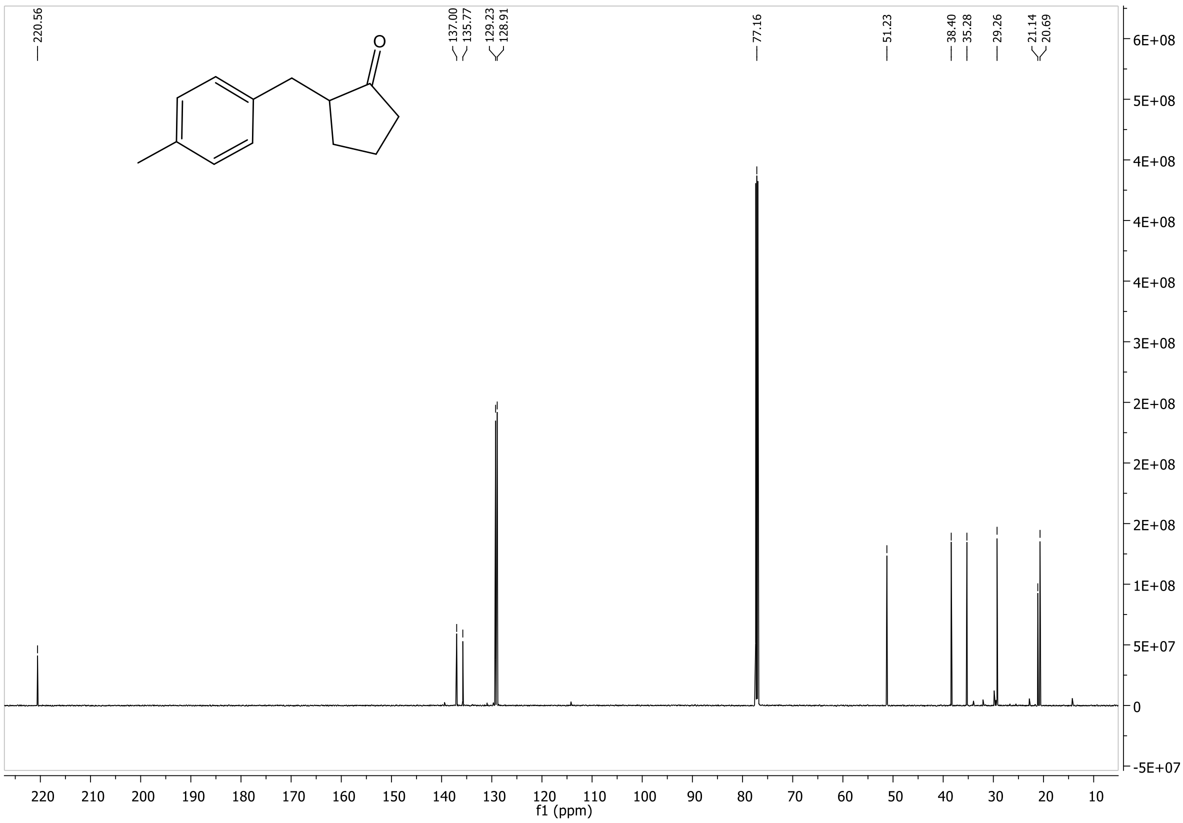

Figure S3: ^{1}H-NMR and ^{13}C-NMR spectrum of ketone 3.

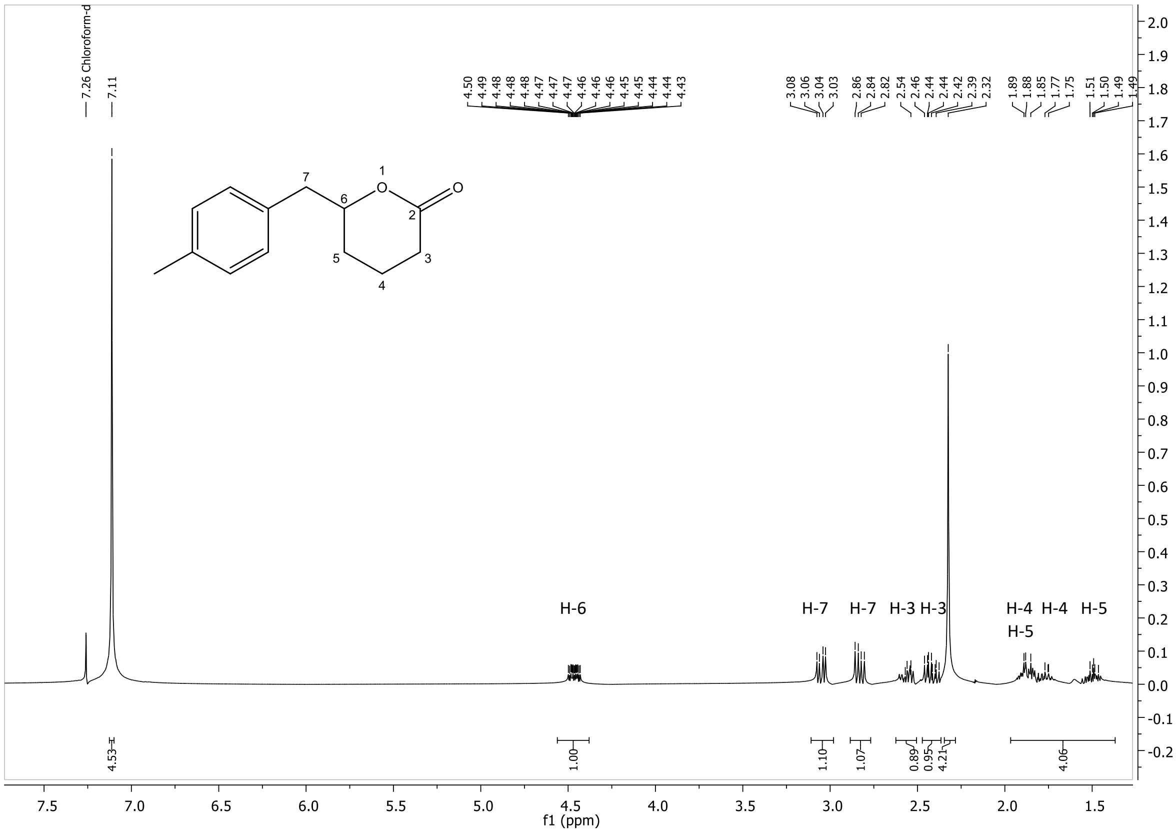

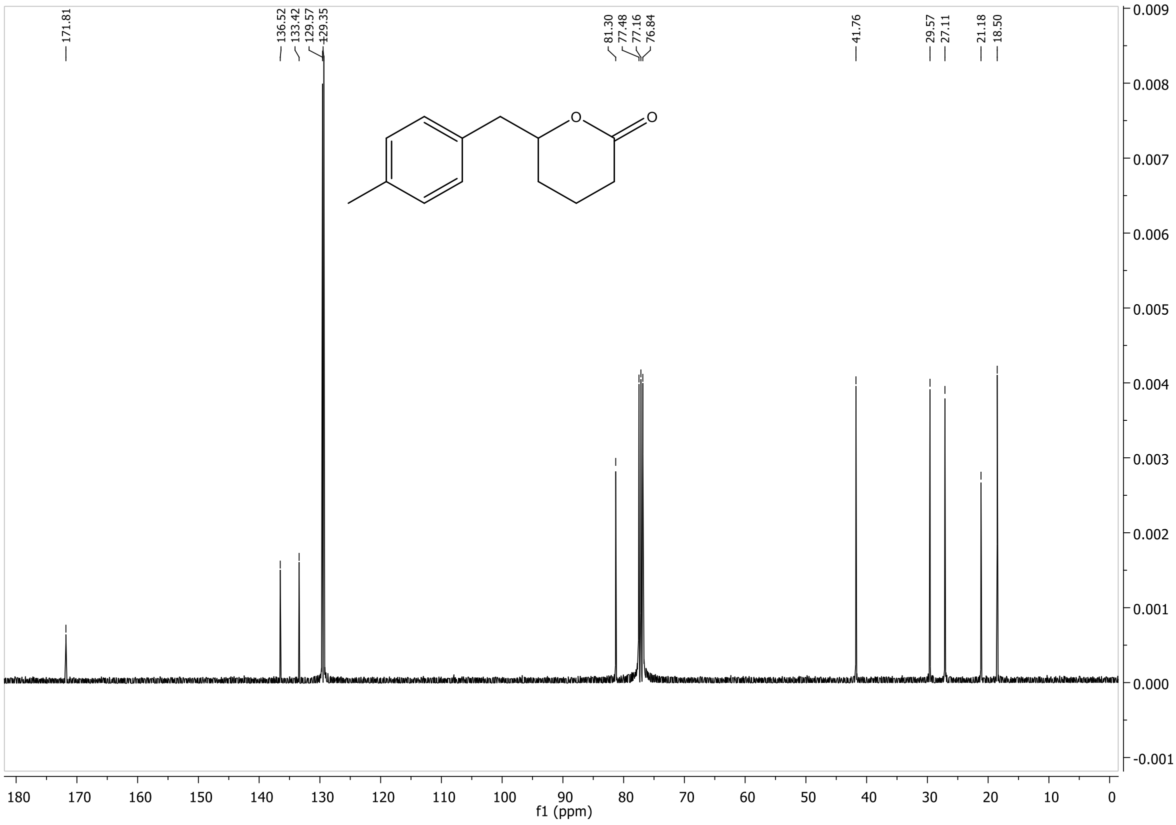

Figure S4: ^{1}H-NMR and ^{13}C-NMR spectrum of lactone 4.

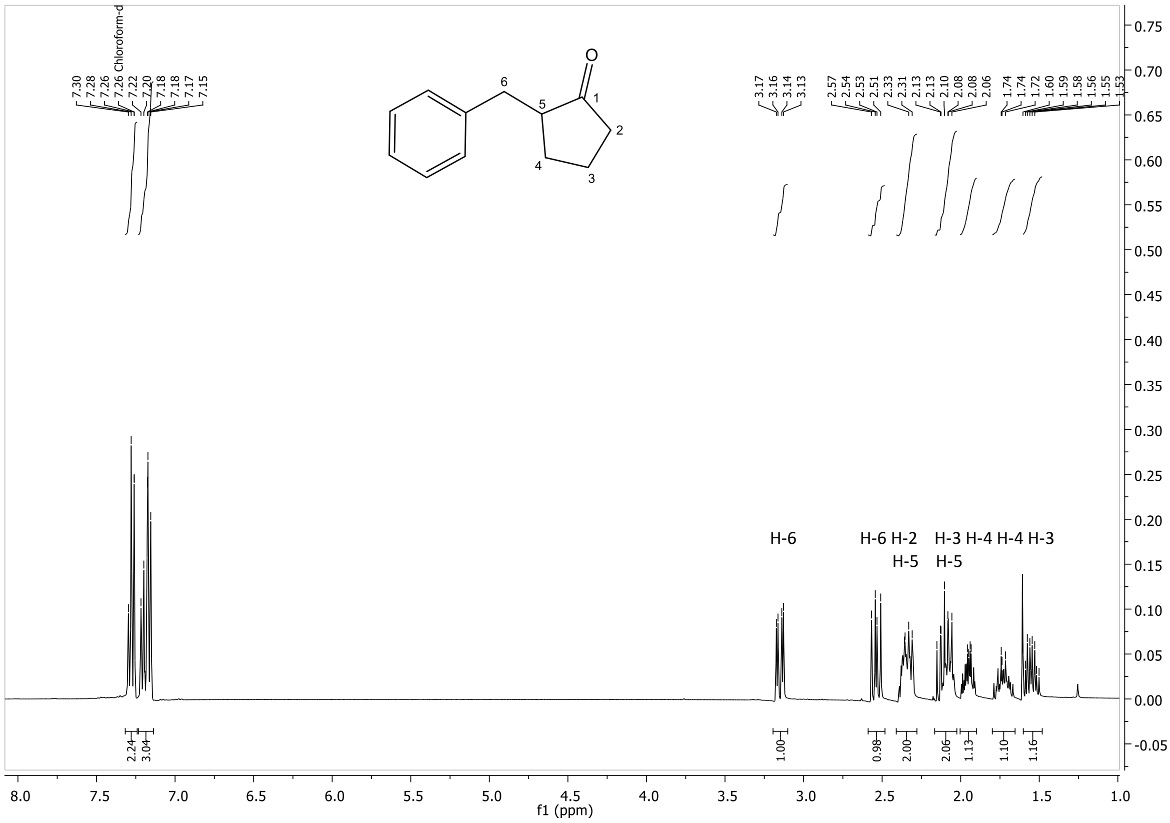

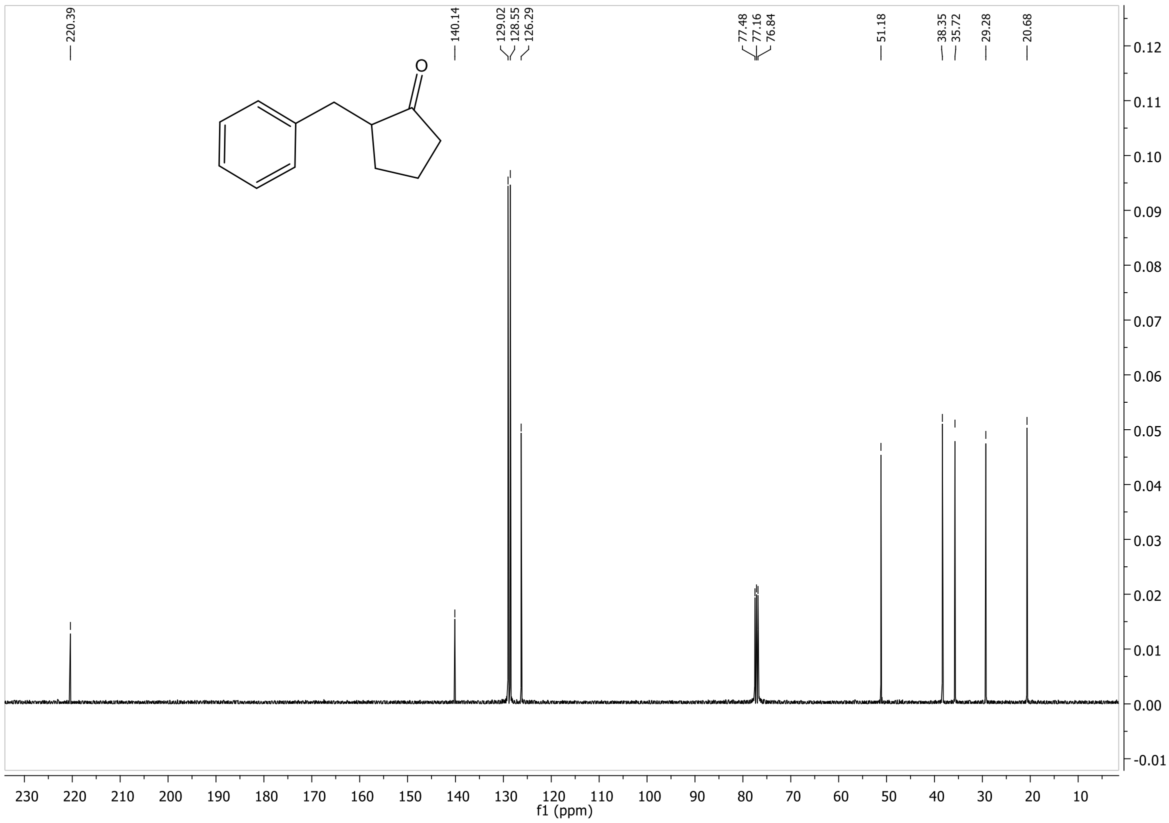

Figure S5: ^{1}H-NMR and ^{13}C-NMR spectrum of ketone 5.

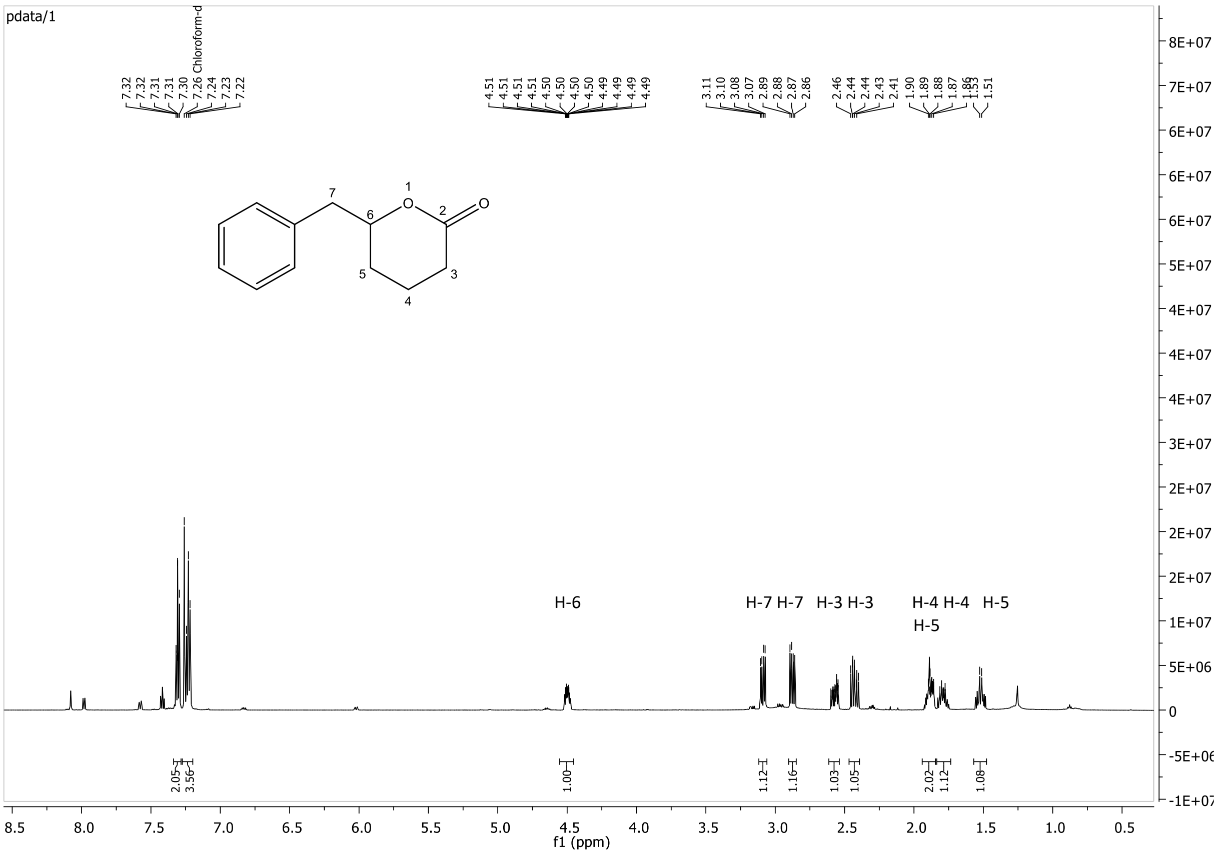

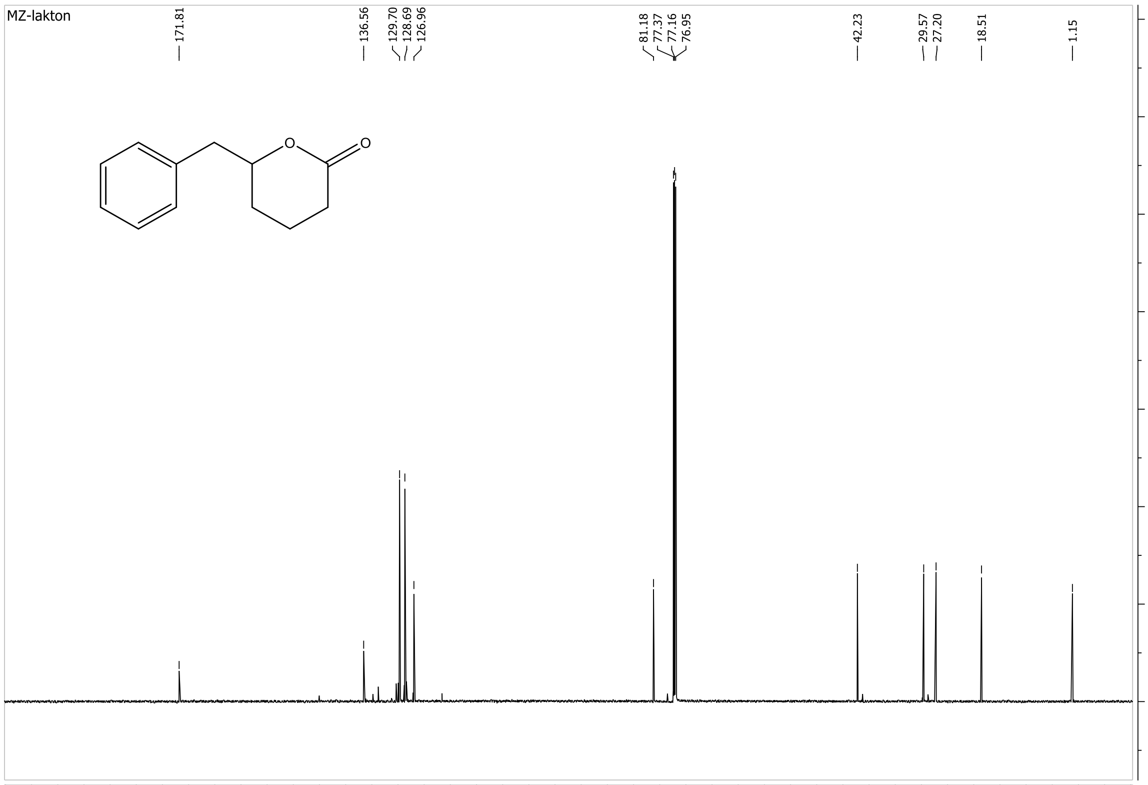

Figure S6: ^{1}H-NMR and ^{13}C-NMR spectrum of lactone 6.

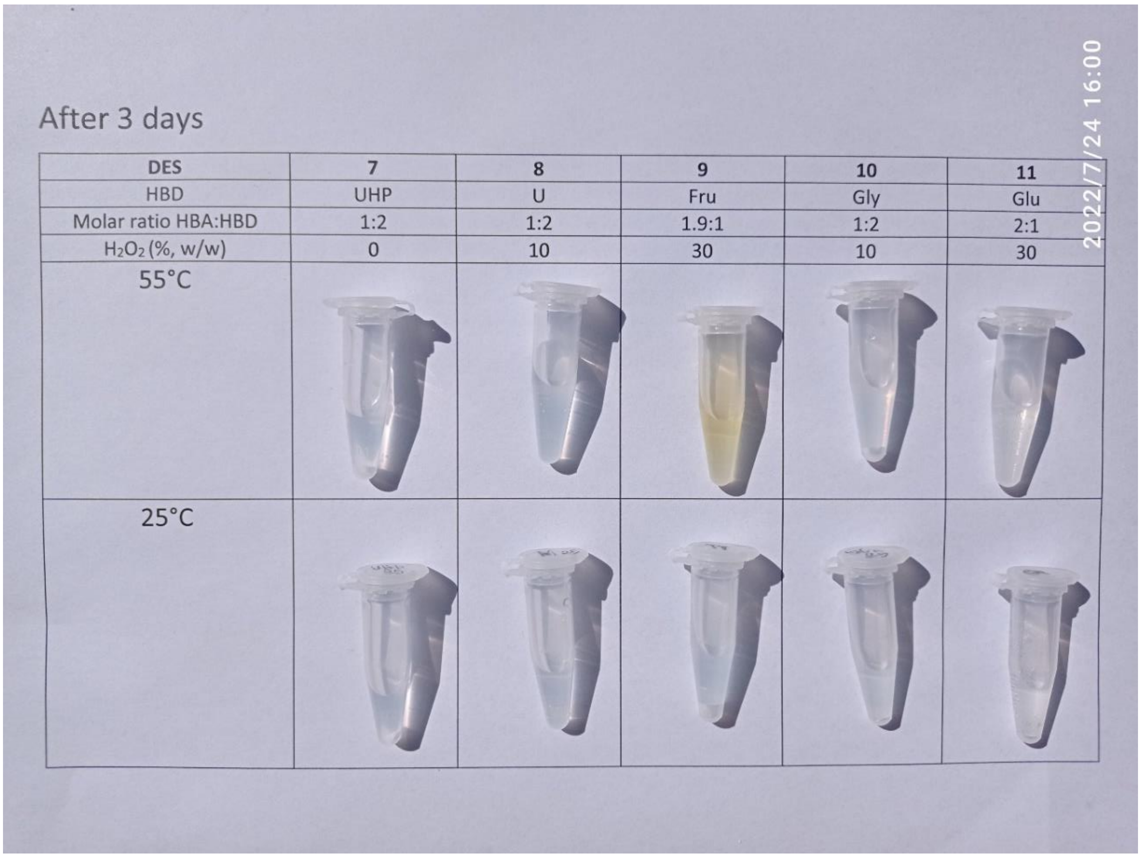

Figure S7. The DESs after 3 days at 25 and 55°C.

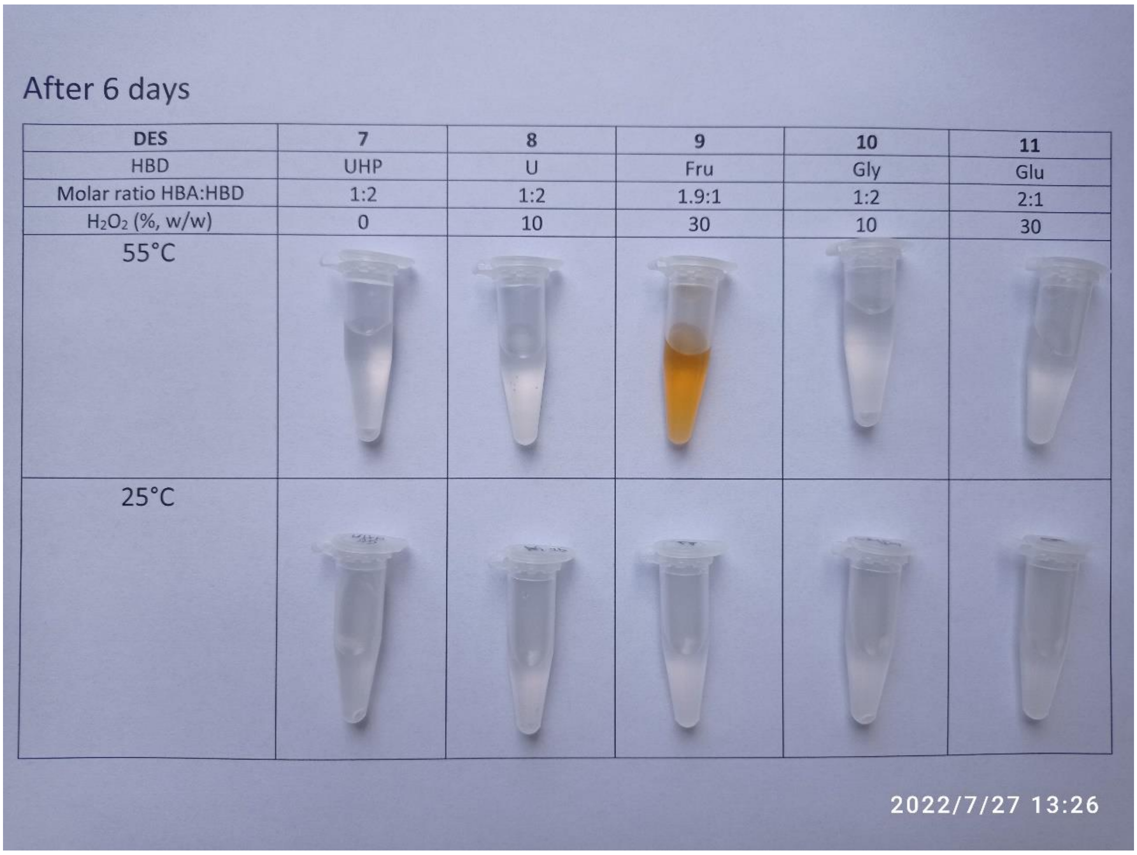

Figure S8. The DESs after 6 days at 25 and 55°C.

Table S1. Comparison of literature data showing BV-type oxidation of cyclic ketones

Entry	Substrate	Product	Solvent	Conversion	Time	References
1	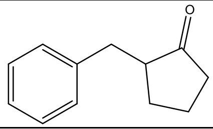	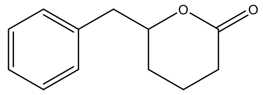	ethyl acetate	93%	5 days	(Ríos et al. 2007)
2	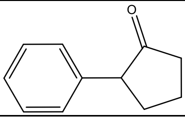	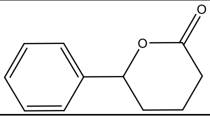		94%	3 days	
3	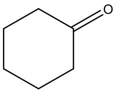	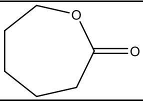	DES - glucose: fructose: sucrose: water (1:1:1:11)	74%	64 h	(Vagnoni et al. 2021)
4	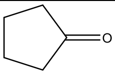	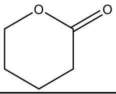		15%	20 h	
5	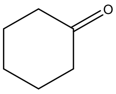	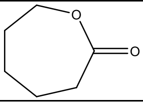	DES - ChCl: sorbitol (1:1)	92%	48 h	(Wang et al. 2017)
6	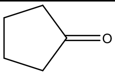	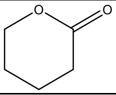		95%	48 h	

Ríos MY, Salazar E, Olivo HF (2007) Baeyer–Villiger oxidation of substituted cyclohexanones via lipase-mediated perhydrolysis utilizing urea–hydrogen peroxide in ethyl acetate. Green Chem 9:459–46. https://doi.org/10.1039/b618175a

Vagnoni M, Samorì C, Pirini D, et al (2021) Lipase catalysed oxidations in a sugar-derived natural deep eutectic solvent. Biocatal Biotransformation 0:1–10. https://doi.org/10.1080/10242422.2021.1913126

Wang XP, Zhou PF, Li ZG, et al (2017) Engineering a lipase B from Candida antactica with efficient perhydrolysis performance by eliminating its hydrolase activity. Sci Rep 7:1–5. https://doi.org/10.1038/srep44599

Table S2. The list of chemicals used in the experiments

	Compound	structure	purity	origin
1	Octanoic acid	$CH_3(CH_2)_6COOH$	≥99%	Sigma–Aldrich
2	urea	$CO(NH_2)_2$	98%	
3	urea hydrogen peroxide (UHP)	$CO(NH_2)_2 \cdot H_2O_2$	97%	
4	hydrogen peroxide	H_2O_2	30 wt. % solution in water	
5	Choline chloride	$(CH_3)_3N(Cl)CH_2CH_2OH$	99%	Acros organics
6	Fructose	$C_6H_{12}O_6$	99%	Alfa Aesar
7	glucose	$C_6H_{12}O_6$	Analytical grade	P.P.H. Stanlab
8	glycerol	$HOCH_2CH(OH)CH_2OH$		Chempur
9	sodium hydrogen carbonate	$NaHCO_3$		Chempur
10	sodium thiosulphate	$Na_2S_2O_3$		Chempur
11	anhydrous magnesium sulphate	$MgSO_4$		POCH
12	Ethyl acetate	$CH_3C(O)OCH_2CH_3$		P.P.H. Stanlab
